# The reproductive biology of *Dyckia scrutor* (Bromeliaceae): an endangered species endemic to Campos Rupestres

**DOI:** 10.1007/s10265-026-01710-7

**Published:** 2026-04-21

**Authors:** Marsal Danrlei de Amorim, Luis Gustavo de Sousa Perugini, Ana Carolina Pereira Machado, Sabrina Aparecida Lopes, Rodrigo Santiago, Lucas Benício de Castro, Bárbara Aparecida Lopes Coelho, André Rodrigo Rech

**Affiliations:** 1 Programa de Pós-Graduação em Ecologia, Conservação e Manejo da Vida Silvestre, Instituto de Ciências Biológicas, Universidade Federal de Minas Gerais, Belo Horizonte, Minas Gerais Brazil; 2 Programa de Pós-Graduação em Ciência Florestal, Universidade Federal dos Vales do Jequitinhonha e Mucuri, Diamantina, MG Brazil; 3 Programa de Pós-Graduação em Biologia Animal, Universidade Federal dos Vales do Jequitinhonha e Mucuri, Diamantina, MG Brazil; 4 Graduação em Ciências Biológicas, Universidade Federal dos Vales do Jequitinhonha e Mucuri, Diamantina, MG Brazil; 5 Instituto de Biología, Pontificia Universidad Católica de Valparaíso, Valparaíso, Chile; 6 Instituto de Biociências, Universidade Estadual Paulista “Júlio de Mesquita Filho”, Rio Claro, São Paulo Brazil; 7 Institute of Entomology, Biology Centre, Czech Academy of Sciences, České Budějovice, Czech Republic; 8 Programa de Pós-Graduação em Ecologia, Evolução e Biodiversidade, Instituto de Biociências, Universidade Estadual Paulista “Júlio de Mesquita Filho”, Rio Claro, São Paulo Brazil

**Keywords:** Bat pollination, Diurnal pollination, Nocturnal pollination, Plant-hummingbird interaction, Threatened species

## Abstract

Pollinators are crucial for the conservation of endangered species, providing essential ecosystem services that support plant reproduction. *Dyckia scrutor* is an endangered species of bromeliad endemic to *Campos Rupestres*. The genus *Dyckia* presents species pollinated by both nocturnal and diurnal visitors, with bats and hummingbirds as the most important pollinators. However, the few existing studies about *D. scrutor* make it difficult to take action to preserve the species. This study aims to describe the floral biology of *D. scrutor* and identify the contribution of nocturnal and diurnal pollinators to the species’ reproductive success. We monitored reproductive phenology, floral longevity, nectar dynamics, floral visitors, pollinator dependence, pollen limitation, and the relative contribution of nocturnal and diurnal visitors. Although the plant produces seeds in the absence of pollinators, their presence increases production. *D. scrutor* showed some degree of pollen limitation, and the main floral visitors were the hummingbird *Augastes scutatus* and *Strymon* butterflies. Although we observed no nocturnal visits, the number of seeds in flowers open to nocturnal pollinators was higher than in those bagged, indicating that nocturnal pollination occurs. However, the contribution of daytime visitors to seed formation was greater. Our work highlights a case of doubly threatened interaction, in which an endangered plant species of restricted range appears to rely primarily on a hummingbird that is also endemic. These results suggest the need for special and cautious measures to preserve this interaction and consequently, the species involved.

**Supplementary Information:**

The online version contains supplementary material available at 10.1007/s10265-026-01710-7.

## Introduction

Since about 79% of all Angiosperms require flower visitors for reproduction (Ollerton et al. 2011; Rodger et al. 2021), the current, ongoing decline of pollinators can affect the diversity of plant species (Potts et al. 2010). These effects could be stronger in plant species with more specialized pollination systems, such as Bromeliaceae, with a close relationship with hummingbird pollination (Santana and Machado 2010). Bromeliaceae A.Juss. is a plant family distributed all over the Neotropical region (Cnc Flora 2023; Gouda et al. 2023). There are more than three thousand bromeliads species, most native to Brazil, specifically to two global hotspots, *Cerrado* and the Atlantic Forest (Cnc Flora 2023). Along with its high diversity, Bromeliaceae has the highest number of critically endangered species in Brazil (Martinelli and Moraes 2013), most of which are threatened by habitat loss and illegal extractivism (Cnc Flora 2023; Martinelli and Moraes 2013). The impact of habitat loss increases for non-studied species with restricted distribution i.e., endemics (Martinelli and Moraes 2013). Regarding pollination, Bromeliaceae is among the few families in which vertebrate pollination predominates over pollination by invertebrates (Santana and Machado 2010; Sazima et al. 1996). In the Atlantic Forest, bromeliads represent more than 30% of the food resources used by hummingbirds (Buzato et al. 2000; Sazima et al. 1996). Besides an intrinsic importance to pollinators, bromeliads are often self-compatible and may produce fruit without pollinators (Christianini et al. 2013; Matallana et al. 2010).

**Fig. 1:**
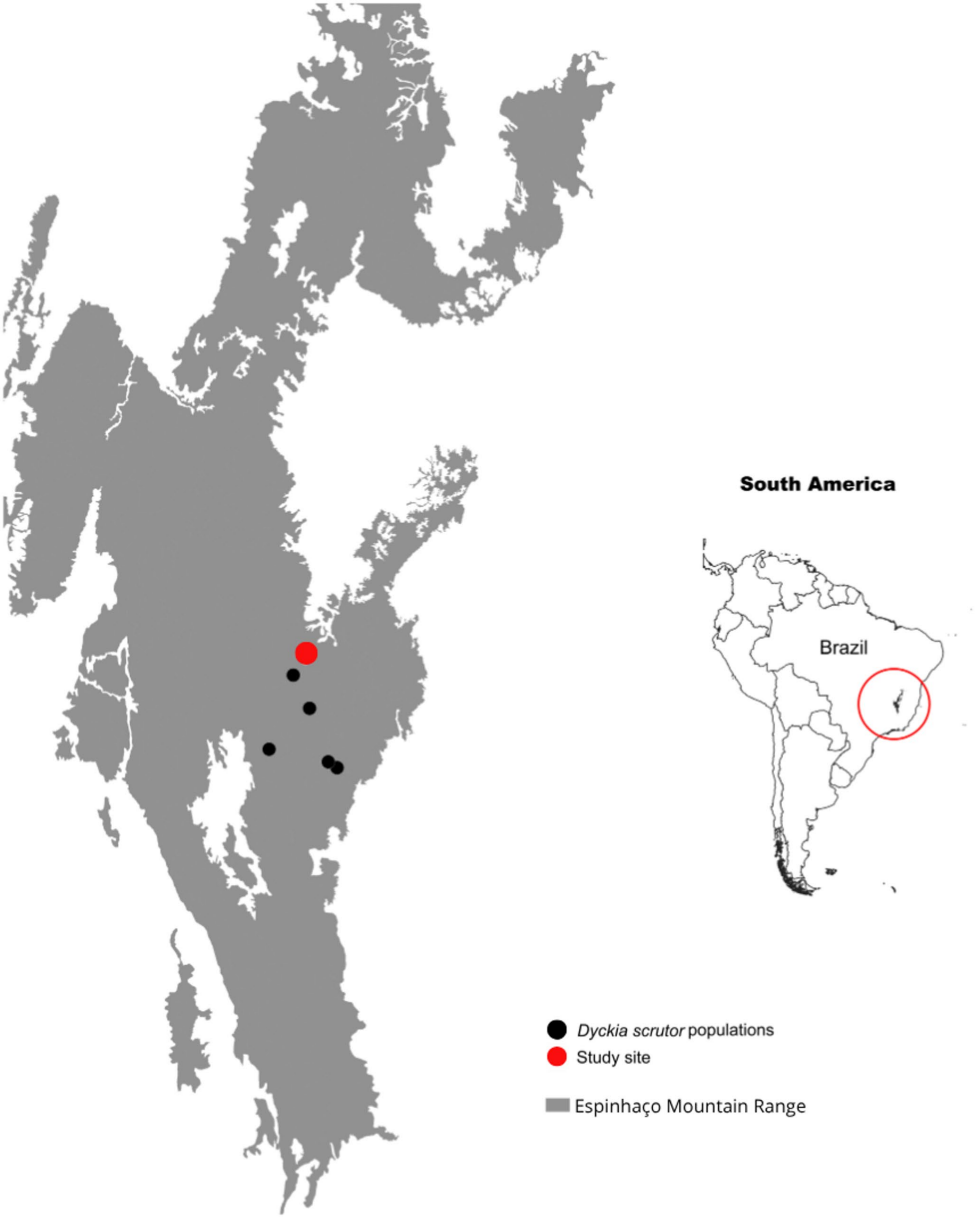
Study site with a new occurrence of *D. scrutor*, and occurrence sites that illustrate a restricted area and endemism. On the right side, inside the red ellipse, is the Espinhaço Mountain Range. On the left side, in gray is the Espinhaço Mountain Range, black dots are the occurrence sites of *D. scrutor* and the red dot represents the area of study

*Dyckia* is a genus of Bromeliaceae, occurring in Brazil, Argentina, Bolivia, and Paraguay. According to the latest taxonomic treatment, it includes species previously classified as *Encholirium, Deuterocohnia, and Dyckia* (Gomes-da-Silva et al. 2019). The genus has a restricted distribution, generally in rocky outcrops and shallow soils (Cnc Flora 2023; Gomes-da-Silva et al. 2017). *Dyckia scrutor* (L.B.Sm.) Rauh (previously: *Encholirium scrutor* L.B.Sm.) is a creeping herb with green petals and pink sepals endemic to the *Campos Rupestres*, a phytophysiognomy of the Cerrado biome, and is listed as Endangered in the Brazilian Red List (Cnc Flora 2023; Colli-Silva et al. 2019; Fig. 1). Its areas of occurrence are within the limits of the Serra do Espinhaço Biosphere Reserve, a biodiversity hotspot (Myers 1990) and a center of endemism for *Dyckia* (Andrade et al. 2018; Cavallari et al. 2006; Forzza et al. 2003). This genus has been reported to be pollinated by bats, hummingbirds, and opossums. However, the pollination system of *D. scrutor* remains unknown (Cristianini et al. 2012; Lenzi and Paggi 2020; Queiroz et al. 2016; Sazima et al. 1989).

The *Campos Rupestres*, or neotropical savannahs, of Minas Gerais belong to the *Cerrado* domain (Bitencourt and Rapini 2013; Silveira et al. 2016) and are characterized as OCBIL (old, climatically-buffered, infertile landscape; Monteiro et al. 2021). The region presents a high diversity of endemic plant species with restricted distributions (Bitencourt and Rapini 2013; Silveira et al. 2016), which can be explained by the accumulation of ancient species, low extinction rates, recent speciation (Rapini et al. 2008; Silveira et al. 2016) and the archipelago vegetation system with rocky outcrops surrounded by sandy fields (Vasconcelos et al. 2020). The generalization of pollination, and therefore the sharing of pollinators, is common in plants from *Campos Rupestres* (Lopes et al. 2022; Maruyama et al. 2013). Even though this strategy may have negative effects (Ashman et al. 2020), *Campos Rupestres* plants seem to benefit from flower visitor sharing, with an increase in their reproductive success (Lopes et al. 2022).

However, anthropogenic threats such as open-pit mining, extensive and recurrent burning and urbanization have led to many of these species, restricted to Brazilian *Campos Rupestres*, to be classified as endangered (Silveira et al. 2016). The pollination biology of *D. scrutor* remains mostly unknown, which hinders the design of adequate conservation strategies for the species (Andrade and Freitas 2021; Faegri and Van der Pijl 1979). The small area of occurrence and its conservation status make the investigation of the reproductive biology of this species urgent (Cnc Flora 2023). The robust floral morphology (tubular corolla) and color (green flowers with red sepals) of *D. scrutor* hint at either hummingbirds or bats as effective pollinators. Therefore, the aim of this study was to understand the floral biology of *D. scrutor*, describing the dynamics of nectar production, its floral visitors, pollinator dependence, pollen limitation and the relative contribution of diurnal and nocturnal visitors to pollen deposition and reproductive success. We also sought to assess whether the reproductive process is in some way among the causes of the Endangered status of the species.

## Materials and methods

### Study area

We collected data between September and December 2022, in a 50-ha area of *Campos Rupestres* located on the JK campus of the Universidade Federal dos Vales do Jequitinhonha e Mucuri, in Diamantina, MG (18°11′ 48.23″ S, 43° 34′ 8.74″ W; Fig. 1). The local climate is classified as mesothermal (Cwb) according to the Köppen–Geiger system, characterized by a humid temperate climate with dry winters (April to September) and moderately hot, rainy summers (October to March) (Alvares et al. 2013). *Campos Rupestres* are a highly heterogeneous environment, with vegetation composed mainly of herbaceous and shrubby plants (Conceição and Pirani 2007; Rapini et al. 2008).

### Reproductive phenology and floral morphology

The reproductive phenology of *D. scrutor* was described by monitoring 30 individuals weekly, from the flower bud stage to the onset of fruiting (between September 24, 2022, and January 6, 2023). For each individual, we recorded the number of flowers in each of these four phenophases: flower bud, flower at anthesis, senescent flower, and onset of fruiting (Bencke and Morellato 2002). To describe floral morphology, we measured width of corolla opening and corolla length, filament (male reproductive structure) and style (female reproductive structure) height, as well as anther and stigma dimensions, using one flower from each of ten individuals (*n* = 10).

### Flower visitor

To describe diurnal and nocturnal floral visitors, we made camera observations (HDR-CX405, SONY, Tokyo) during the day, and visual focal observations at night for 30-min periods (Naqvi et al. 2022). During visits, we noted if the animals touched both reproductive parts (stigma and anther) to confirm their potential effectiveness as pollen vectors for the species under study.

### Nectar dynamics

For nectar dynamics, we bagged flower buds and upon anthesis measured the volume and sugar concentration every 24 h until senescence in different flowers, using a micro-syringe and a handheld refractometer (VODEX, Model VX0-90, Brazil). We used at least four flowers from different individuals for each day of anthesis (from the first to the four days). We tested whether there was an accumulation of nectar volume (response variable) between 2 to 4 days (predictor variable) of flower anthesis using a function “lm” of R-base and “Anova” from the package *Car* (Fox and Weisberg 2019). In this calculation, we used time as a continuous variable. We tested whether nectar concentration (response variable) varies between 2 to 4 days (predictor variable) using a function “lm” of R-base and “Anova” from the package *Car* (Fox and Weisberg 2019). For this calculation, the day was a discrete variable.

### Pollen deposition and removal among diurnal and nocturnal visitors

To measure the removal of pollen from the anthers by floral visitors, we first counted the total amount of pollen produced in anthers still closed in pre-anthesis flower buds. This estimate was later used to compare the pollen count in anthers that were exposed to nocturnal (*n* = 13 anthers) and diurnal (*n* = 14 anthers) floral visitors. We removed the pollen grains from the anthers by placing them in a microtube with 0.2 ml of alcohol. The anthers were macerated in the microtube, the contents homogenized, and a 0.05 ml aliquot transferred to a Neubauer chamber. Five quadrants were counted in the chambers (A, B, C, D and E) and the amount of pollen present in the anthers was estimated by calculating: (number of pollen grains in the five quadrants/0.05 ml present in the chamber) × total volume of solution (0.2 ml). Thus, the amount of pollen removed is the difference between the pollen present in the anthers without visitors and the amount of pollen in the anthers of flowers exposed to visitors. To compare pollen removal between day and night periods, a *t*-test from R-base was used between the amount of pollen removed by day period (response) and the time of day, diurnal and nocturnal (predictor).

To differentiate pollen deposition between diurnal and nocturnal visitors, flowers were left exposed to floral visitors during each period (*n* = 14 diurnal and 13 nocturnal stigma). For this, we bagged flower buds at the beginning of anthesis. For the daytime treatment, we unbagged flowers at 4:00 and bagged them again at 20:00, a period with sunlight, whereas for the night treatment, we unbagged flowers at 20:00 and bagged them at 4:00, a period without sunlight, on the following day. Each flower was exposed to visitors for two periods, two days or two nights. After exposure to diurnal or nocturnal visitors, we collected flowers and made slides of the stigmas in the laboratory using glycerin gelatine stained with fuchsin to make pollen grains evident. We analyzed slides, quantified, and classified the pollen grains as conspecific and heterospecific in both periods (day and night). We tested whether there is a difference between conspecific and heterospecific pollen deposition and pollen richness (response variable) between the nocturnal and diurnal periods (predictor), using three generalized linear models, one for each response variable with a Poisson distribution with the “glm” function of R-base. We ran an additional test for a difference in the composition of pollen types between the two periods (night and day), namely the Fisher’s Exact Test (R core team 2025). To this extra model, we used nine flowers that received heterospecific pollen during the diurnal period and eight flowers that received heterospecific pollen during the nocturnal period.

### Effects of pollination treatments and activity period on reproductive success

To understand the reproductive patterns of *D. scrutor*, we performed reproductive experiments to assess pollinator dependence, pollen limitation, and the relative contribution of diurnal and nocturnal pollinators. We marked 24 flowers from ten individuals to determine whether the species produces seeds through spontaneous self-pollination. In addition, 22 flowers from ten individuals were exposed to pollinators to evaluate seed production under natural pollination, and 19 flowers from ten individuals were used for manual cross-pollination. The manual cross-pollination consisted of adding pollen from the anthers of flowers belonging to different individuals of the same species onto the stigma of experimental flowers. For this, we used the anthers of three flowers from different individuals as pollen donors. To assess the relative contribution of diurnal and nocturnal pollinators, we marked flowers to be exposed to pollinators separately during each period (daytime and nighttime), at the same times used for measuring pollen deposition and removal in the previous section. For these two treatments, we bagged 11 flowers to estimate pollination during the nocturnal pollination and eight flowers for diurnal pollination. These flowers were exposed for two consecutive days, corresponding to two nights or two days. After fruit development, the flowers were collected and their seeds were counted. To test for differences among treatments, we used a generalized linear model with a Poisson distribution, implemented with the function “glm” from the base R package, followed by Tukey’s post hoc test using the emmeans package for pairwise contrasts among treatments (Lenth 2025). All analyses were performed in R version 4.5.1 (R core team 2025), using R-Studio (Posit Team 2025).

## Results

### Reproductive phenology and floral morphology

**Fig. 2:**
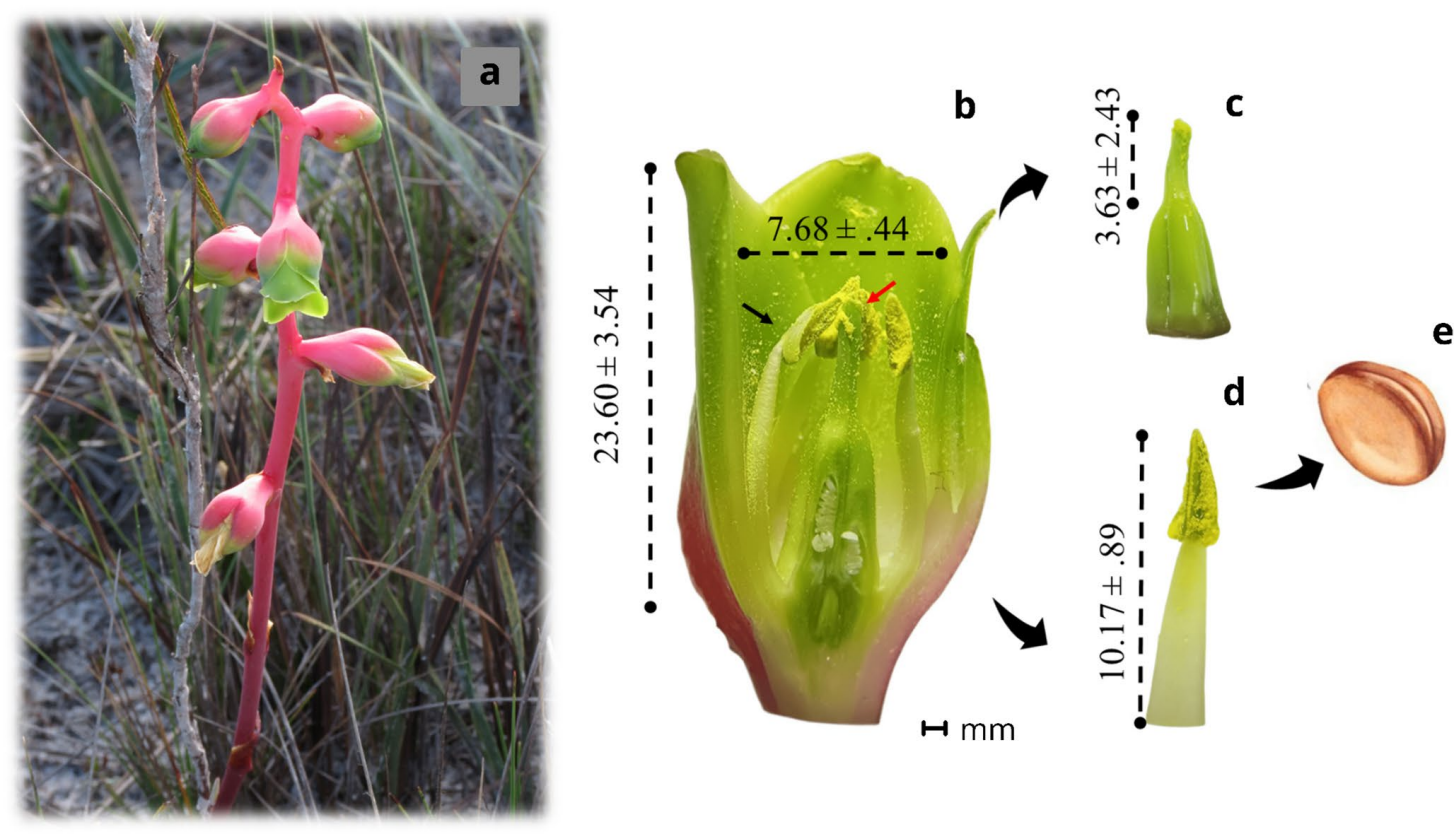
Floral structures of *D. scrutor* with mean ± standard deviation (in millimeters). **a** Individuals of *D. scrutor* show senescent flowers, open flowers, and bud flowers from the bottom to the top; **b** Corolla opening and length. Position of the anther (black arrow) and stigma (red arrow) in the flower; **c** Stigma; **d** Anther; **e** Pollen grain illustration of the *D. scrutor*

In the population studied, we found 104 individuals of *D. scrutor* with a total of 143 flowers. In general, individuals had only 1 or 2 flowers blooming simultaneously. The flowers of *D. scrutor* are odorless to humans and the anthers cover the stigmas (Fig. 2). The average flower longevity is approximately five days. Monitoring the population’s reproductive phenology showed that five weeks elapsed between the bud stage and the start of fruiting (Fig. S1). We recorded open flowers from the first week to the third, with a peak of flowering intensity in the first week (Fig. S1).

### Flower visitor

**Fig. 3:**
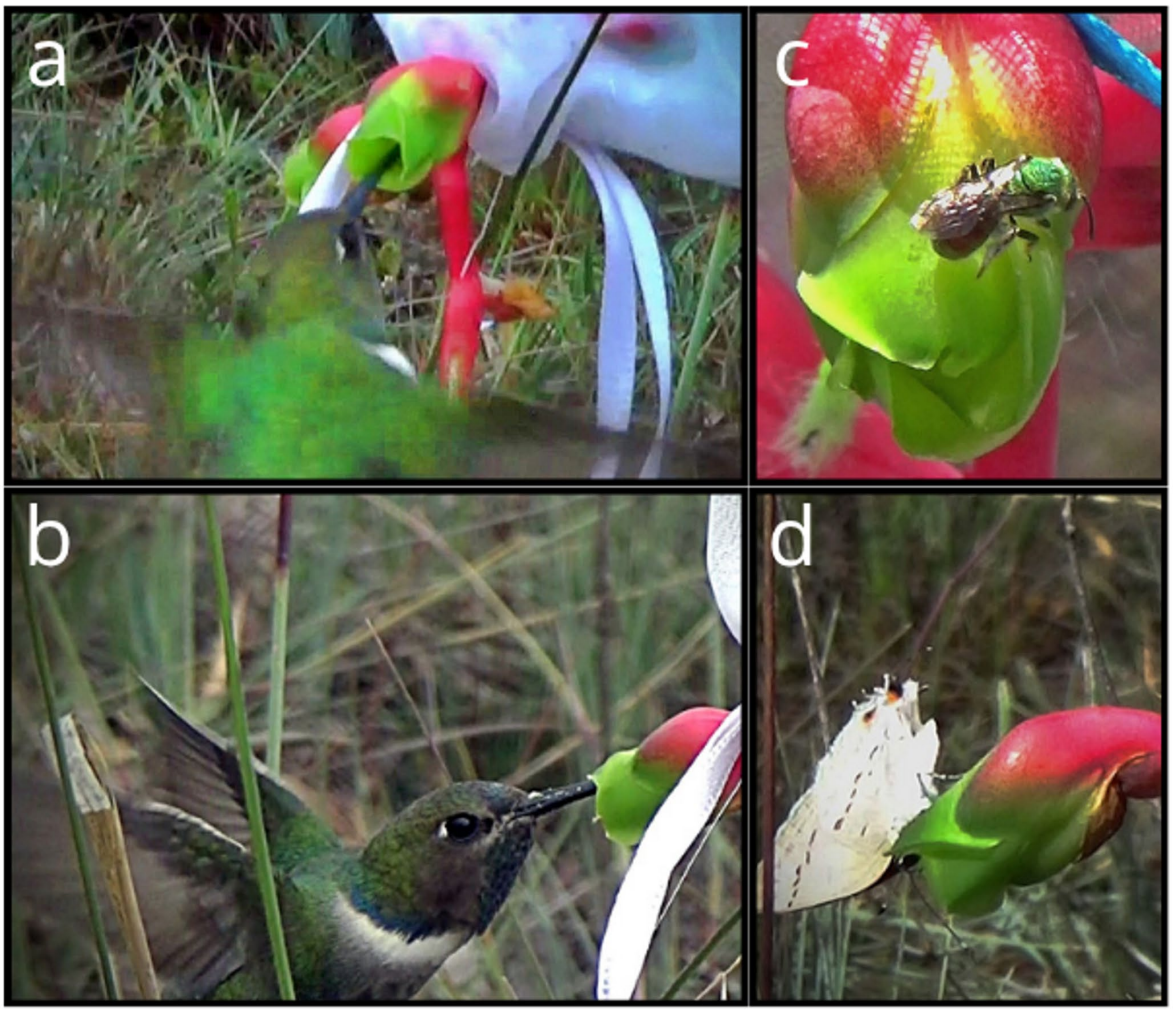
Pollinators and flower visitors of *D. scrutor*. **a** and **b** the hummingbird *A. scutatus*, **c** a bee from the Halictidae family, and **d** the butterfly *Strymon* sp

A total of 30 visits were observed in 30 h of observation, with 24 h during the day and 6 h at night (Fig. 3). Five species of ant and the butterfly *Strymon* sp. were the most frequent visitors, with 18 and 6 visits, respectively. Only *Strymon* sp. and the hummingbird *Augastes scutatus* (three visits) touched the reproductive parts and were therefore considered potential pollinators. An unidentified bee with one visit and one Halictidae bee with two visits, were not observed touching the stigmas, being possible pollen robbers. No visits were observed during the nocturnal observations. The lack of visits during the night could be related to the short observation time.

### Nectar dynamics

**Fig. 4:**
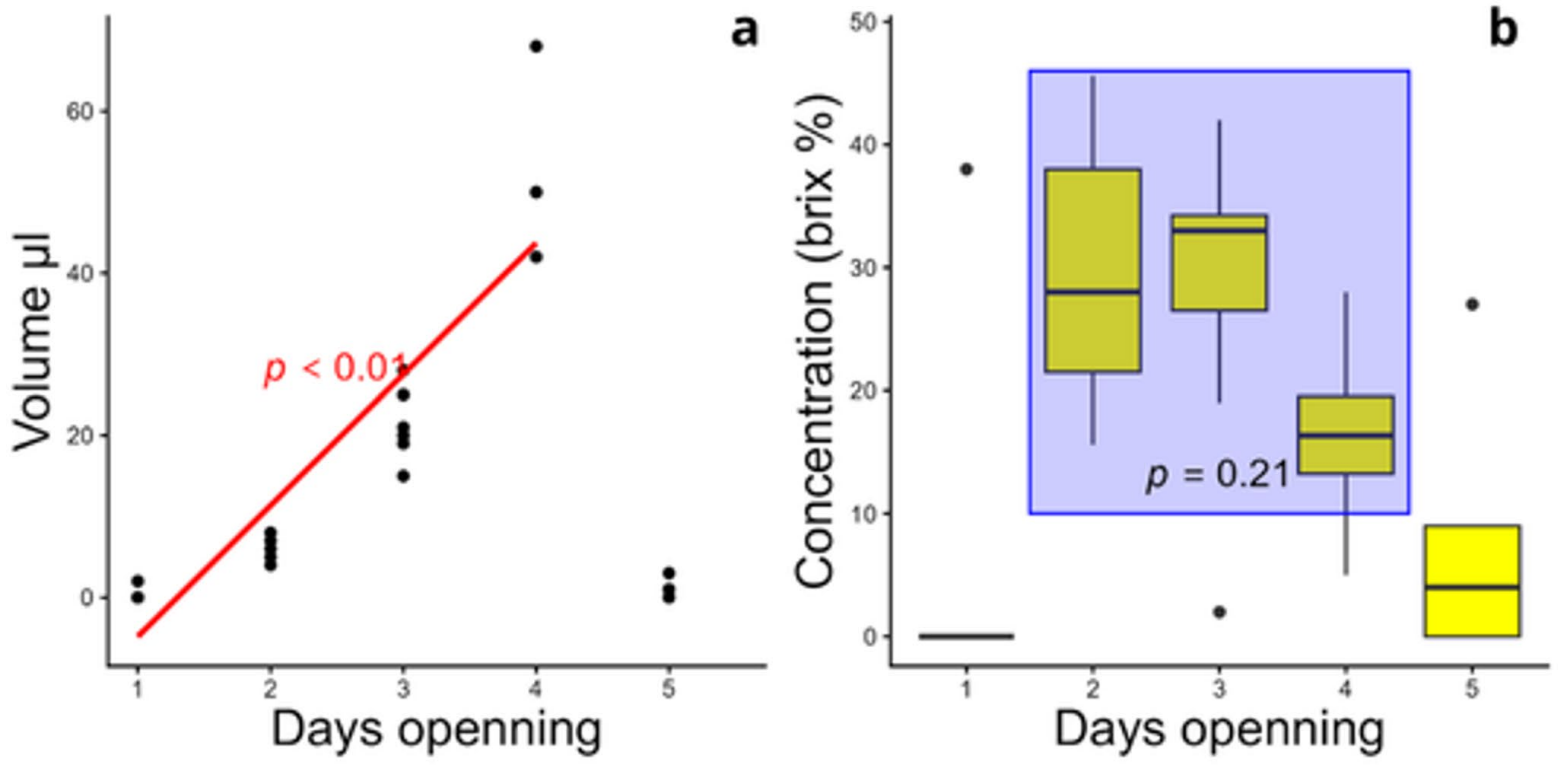
Nectar dynamics of *D. scrutor*. **a** Accumulated volume between the first and fourth day of flower anthesis, and absorption between the fourth and fifth day. **b** Nectar concentration on each day of flower anthesis, between the second and fifth day of anthesis

The dynamics of nectar secretion and reabsorption lasted four days. Nectar became available from the second day (Fig. 4a). There was an accumulation of nectar until the fourth day of anthesis, with a peak volume of between 40 and 60 μL on the fourth day (F_(1,21)_ = 105.99; *p* < 0.01) and reabsorption on the fifth day. There was no variation in concentration, with steady 25.96 (sd = 12.31) °Brix (F_(2,14)_ = 1.73; *p* = 0.21; Fig. 4b). Considering the average production of nectar and concentration and the number of individuals in the local area, the population produced around 2.5 kcal/day.

### Pollen deposition and removal among diurnal and nocturnal visitors

**Fig. 5:**
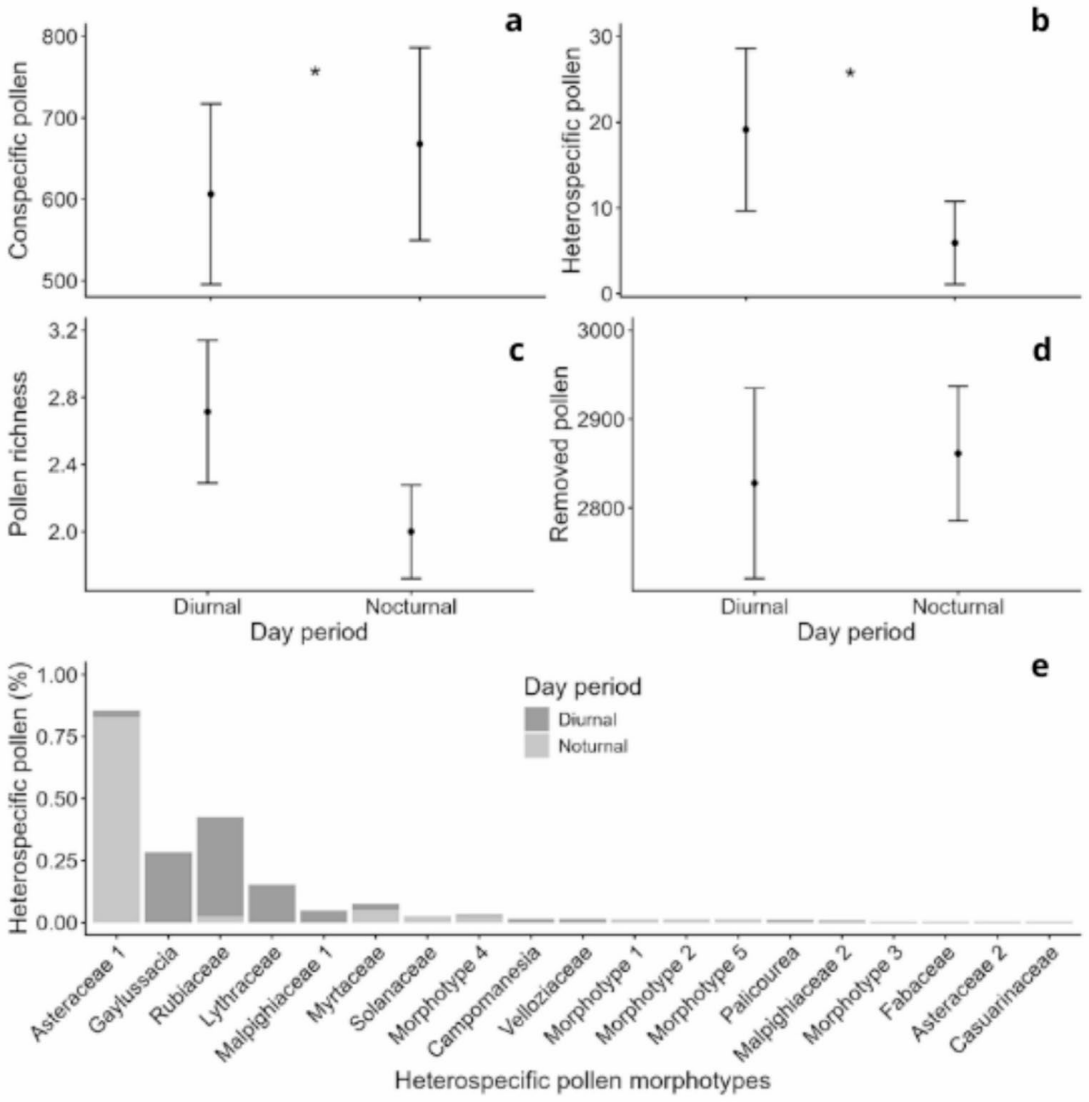
Deposition and removal of conspecific and heterospecific pollen grains on the stigmas of *D. scrutor* flowers. Difference in **a** conspecific pollen deposition, **b** heterospecific pollen deposition, **c** heterospecific pollen richness, and **d** pollen removal from anthers between nocturnal and diurnal visitors. * represents a significant difference. Bars represent the mean ± standard error (SE). **e** Heterospecific pollen load composition and abundance between nocturnal and diurnal flowers

No anther was found depleted of pollen grains and pollen removal did not differ between night and day periods (t = 0.25; df = 25; *p* = 0.59; Fig. 5d). Conspecific pollen deposition varied between 86 and 1476 grains per stigma, with lower deposition during the day than during the night ($\chi$^2^ = 40.23; *n* = 27; *p* < 0.001; Fig. 5a). On the other hand, heterospecific pollen deposition was higher during the day ($\chi$^2^ = 97.45; *n* = 27; *p* < 0.001; Fig. 5b). Heterospecific pollen was found on 17 of the 27 stigmas, nine on diurnal and eight on nocturnal. Heterospecific pollen richness did not differ between night and day periods ($\chi$^2^ = 3.23; *n* = 27; *p* = 0.07; Fig. 5c), but the composition of pollen types found on the stigma was different between the periods (*p* = 0.0004, Fig. 5e). There were around 19 heterospecific pollen types, of which the most recurrent were Rubiaceae sp1, *Gaylussacia* sp. Asteraceae sp1 and Lythraceae sp. (Fig. S2). We also found pollen grains from Casuarinaceae, Fabaceae, Malvaceae, Myrtaceae, Solanaceae and Velloziaceae, as well as genera such as *Palicourea* and *Campomanesia*. Thus, the female success, measured by the deposition of conspecific pollen, was higher at night, while male success, measured by pollen removal, did not differ between day and night.

### Effects of pollination treatments and activity period on reproductive success

We recorded seed set in two fruits out of the 24 flowers bagged to test for spontaneous self-pollination (Table 1). The mean number of seeds resulting from spontaneous self-pollination (3.04 ± 12.4, Average and Sd respectively) was lower than in all other treatments (Table 1). Seed production under natural pollination (7 ± 13) and nighttime-opened flower (5.54 ± 12.01) did not differ significantly from each other (Table 1), but both were lower than seed production in daytime-opened flowers (13.12 ± 21.51) and in manual cross-pollination (12.9 ± 20.4). The latter two treatments did not differ significantly from each other (Table 1).

**Table 1:** Reproductive tests of *D. scrutor* in Campos Rupestres at the JK Campus, Universidade Federal dos Vales do Jequitinhonha e Mucuri, Diamantina, Brazil

Treatment	Sample	Average	SD	Significance
Self pollination	24	3.04	12.4	a
Natural pollination	22	7	13	b
Nighttime-opened flower	11	5.54	12.01	b
Cross pollination	19	12.9	20.4	c
Daytime-opened flowers	8	13.12	21.51	c

Treatment indicates the type of reproductive test applied. Sample is the number of flowers used per treatment. Average represents the mean seed set, and SD is the standard deviation. Different letters indicate significant differences among treatments (Tukey test, *p* < 0.04)

## Discussion

In this study, we have shown that *D. scrutor*, an Endangered bromeliad, can produce autogamous seeds, but the presence of pollinators magnifies its reproductive success (Table 1). The composition of heterospecific pollen loads is different between nocturnal and diurnal visitors, indicating that these visitor groups have different floral preferences (Fig. 5e). We also demonstrated that the species offers nectar as a resource and can be pollinated by nocturnal and diurnal visitors that transfer both conspecific and heterospecific pollen, producing more seeds than in bagged flowers (Fig. 5). Although they deposit less conspecific pollen during the day, diurnal pollinators, represented mainly by the endemic hummingbird *A. scutatus* and the butterfly *Strymon sp.*, seem to be the most important for seed production in *D. scrutor*. The reproductive process of producing seeds of *D. scrutor* is not a constraint factor resulting in its Endangered status.

The ability of *D. scrutor* to produce seeds in the absence of pollinators, as well as the vegetative reproduction found in patches, may contribute to the species’ reproductive security in environments with rare or unpredictable pollinators (Cavallari et al. 2006; Christianini et al. 2013; Fausto et al. 2001). Also, Bromeliaceae are commonly reported to be self-compatible but with deformed seeds generated by self-pollination (Christianini et al. 2013; Matallana et al. 2010). In the presence of pollinators, *D. scrutor* seed production increases significantly (Table 1). This is important because cross-pollination in *Dyckia* species can promote gene flow between populations, and these episodes, along with seed dispersal, can enhance gene diversity between populations (Gonçalves-Oliveira et al. 2017).

Even though they are distributed in a highly heterogeneous environment, plants from *Campos Rupestres* generally have low levels of pollen limitation (Lopes et al. 2023). Interactions with different floral visitors can help reduce pollen limitation, as the sharing of pollinators increases the number of visits and pollen deposition (Lopes et al. 2023; Martén-Rodríguez and Fenster 2010). One factor that generates pollen limitation is the high deposition of heterospecific pollen grains, a phenomenon commonly observed in plants pollinated by hummingbirds (Lopes et al. 2022; Morales and Traveset 2008; Streher et al. 2020). The high deposition of heterospecific pollen on stigmas may be one of the main factors contributing to the high level of pollen limitation in the *D. scrutor* population (Shivanna 2015).

The flowers of *D. scrutor* have a relatively large opening, allowing bees and ants to enter and contact the anthers, thereby increasing the rate of self-pollen deposition. However, bees and ants may not touch the stigmas because the anthers are above the stigmas (Fig. 3). The hummingbird *A. scutatus* and the butterfly *Strymon* sp*.* touch both reproductive parts during their visit and are thus potential pollinators. However, butterfly proboscises are narrower compared to the flower tube. The visiting behavior regarding the corolla opening suggests that *A. scutatus* has the better morphological coupling (Rodríguez-Otero et al. 2023; Vizentin-Bugoni et al. 2014). This hummingbird is known to be a generalist and uses several species, including non-ornithophilous ones, for food (Queiroz 2018; Rodrigues and Rodrigues 2014). In this sense, hummingbirds are not dependent on a single resource.

Although we did not record any visits during the nocturnal observations, not only did the flowers show heterospecific pollen deposition, but the composition of the pollen types was also different during the night period (Fig. 5). These results and the higher number of seeds compared to self-pollination suggest that visitation occurs and that it is effective (Table 1). However, the higher fruit set observed under diurnal pollen deposition, even when compared to natural pollination, was intriguing and suggests that pollen quality may play an important role. Nocturnal pollen deposition may reduce fertilization efficiency or interfere with subsequent diurnal pollen performance. Additionally, the lower visitation frequency of nocturnal pollinators may result in reduced pollen viability by the time pollen reaches the stigma. Nevertheless, further studies are needed to better understand this pattern. Environmental factors such as temperature, humidity, and timing of pollen deposition are known to influence pollen tube growth and fertilization success (Brunet et al. 2019; Dafni and Firmage 2000). Hence, the frequency of the diurnal pollinators moving to another *D.scrutor* individual could be higher than that of the nocturnal pollinators. This difference in the time that conspecific pollen is carried until arrival in the stigmas could influence the viability and vigor, and finally produce more seeds in the day than in the night, despite the pollen quantity. There is a mixed reproductive system, with diurnal and nocturnal visitors occurring in *Dyckia* species (Queiroz et al. 2016). The use of diurnal and nocturnal pollinators increases the generalization of the pollination system and can also be understood as a common reproductive security mechanism in tropical high-altitude environments or islands (Bergamo et al. 2022; Martén-Rodríguez et al. 2010). The plant’s low height relative to the vegetation and its lack of scent can be unfavorable to visitation by bats (Fleming et al. 2009 but see Amorim et al. 2022). Butterfly visits during the day could indicate that moths are nocturnal visitors of *D. scrutor*, due to the morphological similarity between adult nectar-feeding Lepidoptera (Krenn 2010). This similarity, however, does not fit the difference in the composition of heterospecific pollen loads between nocturnal and diurnal.

*Dyckia* species exhibit low seed dispersal (Holst 1994), with most seeds remaining close to the mother plant (Cavallari et al. 2006), contributing to population isolation. Despite their restricted distribution, species of this genus tend to maintain a reasonable level of genetic diversity (Cavallari et al. 2006; Gonçalves-Oliveira et al. 2017, 2020), but this diversity occurs between populations, indicating genetic isolation (Gonçalves-Oliveira et al. 2017; Hmeljevski et al. 2017; Ruas et al. 2020). Long-distance pollination by large bees, hummingbirds, and bats plays a crucial role in connecting populations (Monteiro et al. 2021), acting as the primary driver of gene flow for these species (Cavallari et al. 2006; Gonçalves-Oliveira et al. 2017, 2020). In such cases, even a few cross-pollination events between populations may be sufficient for natural genetic recovery (Slatkin 1985; Wright 1990). *D. scrutor* has an overlapping distribution with *A. scutatus*, its potential main pollinator, which is endemic to the southern Espinhaço Range (Vasconcelos 2008), suggesting that this hummingbird plays a key role in connecting populations.

Currently, the Campos Rupestres face various anthropogenic pressures, including mining, wildfires, and urbanization, which, combined with the high geographic restrictions of Dyckia species, increase extinction risks, especially since many species occur outside protected areas (Versieux and Wendt 2007). This makes it urgent to implement protective measures not only for Dyckia species but also for the Campos Rupestres as a whole (Versieux and Wendt 2007). Protection could be achieved through the establishment of Conservation Units in unprotected habitats (Kearns et al. 1998; Lee et al. 2007), which should also consider interactions with pollinators (Cerceau et al. 2019), given their importance for gene flow between populations and community structure. In addition to Conservation Units, both in situ (Cavallari et al. 2006; Gonçalves-Oliveira et al. 2017; Hmeljevski et al. 2017; Ruas et al. 2020) and ex situ (Cavallari et al. 2006; Rogalski et al. 2017) conservation efforts are necessary; however, caution is needed when removing species from natural populations, as this may significantly impact genetic diversity (Cavallari et al. 2006).

*D. scrutor* is a species endemic to *Campos Rupestres* with an Endangered and a restricted distribution, but a fairly generalist pollination system with diurnal and nocturnal flower visitors. Although the species does not depend on pollinators to produce seeds, it produces more seeds when they are present. Nocturnal visitors deposit more pollen but visits from diurnal pollinators result in more seeds. *Campos Rupestres* form an extremely diverse and still little-known environment that is at risk from anthropogenic threats, which is why studies such as ours are important for creating strategies to preserve this environment and the interactions occurring within it.

## Supplementary Information

Below is the link to the electronic supplementary material.

Supplementary Material 1
