# Supplementary material for: The reproductive biology of Dyckia scrutor (Bromeliaceae): an endangered species endemic to Campos Rupestres
Source: J Plant Res. 2026 Apr 21;139(3):443–54. doi: 10.1007/s10265-026-01710-7 (PMC13197304; doi:10.1007/s10265-026-01710-7)
Supplement: Supplementary file 1 — Supplementary Material 1 [file 10265_2026_1710_MOESM1_ESM.pdf]

Title: The Reproductive Biology of *Dyckia scrutor* (Bromeliaceae): an Endangered Species Endemic to Campos Rupestres

Marsal Danrlei de Amorim[1,6]*; Luis Gustavo de Sousa Perugini[2,7]; Ana Carolina Pereira Machado[2,5], Sabrina Aparecida Lopes[2], Rodrigo Santiago[3], Lucas Benício de Castro[3,8], Bárbara Aparecida Lopes Coelho[4], André Rodrigo Rech[2, 3,4]

[1] Programa de Pós-Graduação em Ecologia, Conservação e Manejo da Vida Silvestre, Instituto de Ciências Biológicas, Universidade Federal de Minas Gerais, Belo Horizonte, Minas Gerais, Brazil.

[2] Programa de Pós-Graduação em Ciência Florestal, Universidade Federal dos Vales do Jequitinhonha e Mucuri, Diamantina, MG, Brazil.

[3] Programa de Pós-Graduação em Biologia Animal, Universidade Federal dos Vales do Jequitinhonha e Mucuri, Diamantina, MG, Brazil.

[4] Graduação em Ciências Biológicas pela Universidade Federal dos Vales do Jequitinhonha e Mucuri, Diamantina, MG, Brazil.

[5]Instituto de Biología, Pontificia Universidad Católica de Valparaíso, Valparaíso, Chile

[6]Instituto de Biociências, Universidade Estadual Paulista "Júlio de Mesquita Filho", Rio Claro, São Paulo, Brazil.

[7]Institute of Entomology, Biology Centre, Czech Academy of Sciences, České Budějovice, Czech Republic.

[8]Programa de Pós-Graduação em Ecologia, Evolução e Biodiversidade, Instituto de Biociências, Universidade Estadual Paulista "Júlio de Mesquita Filho", Rio Claro, São Paulo, Brazil.

* Corresponding author: marsal.amorim@gmail.com

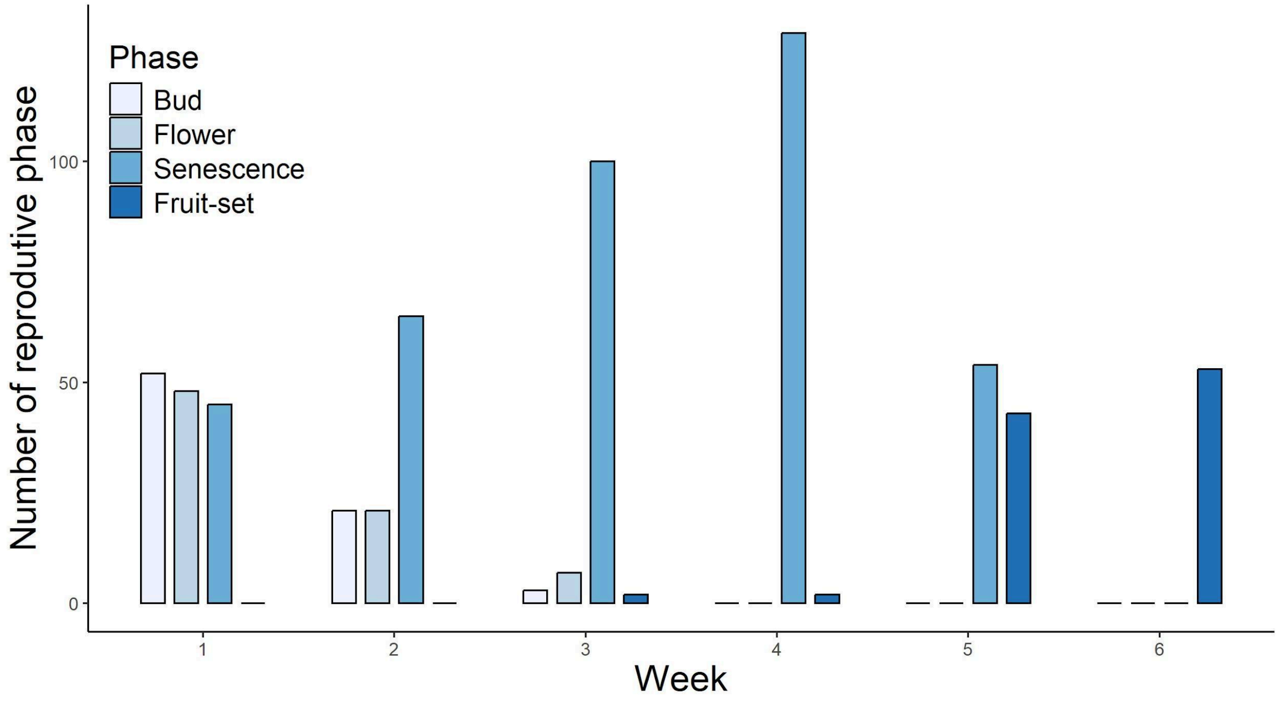

Fig S1 Flowering phenology stages of *D. scrutor* and sampling weeks. The first week of sampling was on 24/11/2023.

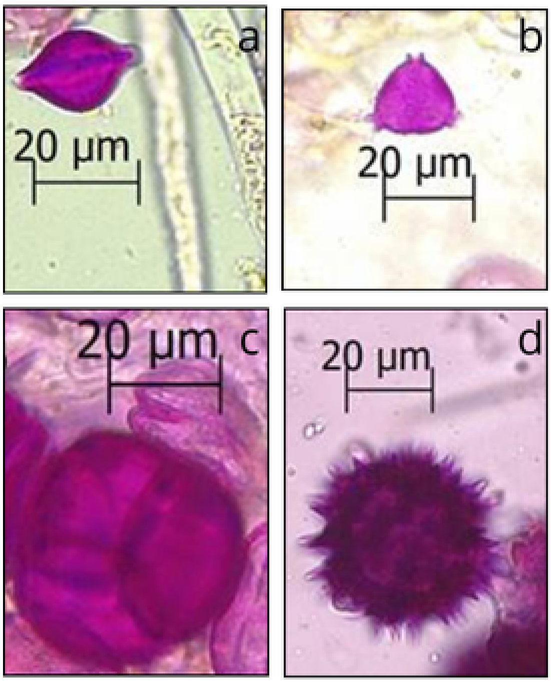

Fig S2 Most recurrent pollen grains found on the stigma of *D. scrutor*. (a) Rubiaceae sp.; (b) Lythraceae sp. (c) *Gaylussacia* sp. and (d) Asteraceae sp1.
